# A statistical framework for detecting mislabeled and contaminated samples using shallow-depth sequence data

**DOI:** 10.1186/s12859-018-2512-8

**Published:** 2018-12-12

**Authors:** Ariel W. Chan, Amy L. Williams, Jean-Luc Jannink

**Affiliations:** 1 Section of Plant Breeding and Genetics, School of Integrative Plant Sciences, Cornell University, 407 Bradfield Hall, Ithaca, NY 14853 USA; 2 Department of Biological Statistics and Computational Biology, Cornell University, Ithaca, NY 14853 USA; 3 RW Holley Center for Agriculture and Health, United States Department of Agriculture -- Agricultural Research Service, School of Integrative Plant Sciences, Cornell University, 258 Emerson Hall, Ithaca, NY 14853 USA

**Keywords:** Error detection, Biological replication, Technical replication, Shallow-depth sequence data, Mislabeled samples

## Abstract

**Background:**

Researchers typically sequence a given individual multiple times, either re-sequencing the same DNA sample (technical replication) or sequencing different DNA samples collected on the same individual (biological replication) or both. Before merging the data from these replicate sequence runs, it is important to verify that no errors, such as DNA contamination or mix-ups, occurred during the data collection pipeline. Methods to detect such errors exist but are often ad hoc, cannot handle missing data and several require phased data. Because they require some combination of genotype calling, imputation, and haplotype phasing, these methods are unsuitable for error detection in low- to moderate-depth sequence data where such tasks are difficult to perform accurately. Additionally, because most existing methods employ a pairwise-comparison approach for error detection rather than joint analysis of the putative replicates, results may be difficult to interpret.

**Results:**

We introduce a new method for error detection suitable for shallow-, moderate-, and high-depth sequence data. Using Bayes Theorem, we calculate the posterior probability distribution over the set of relations describing the putative replicates and infer which of the samples originated from an identical genotypic source.

**Conclusions:**

Our method addresses key limitations of existing approaches and produced highly accurate results in simulation experiments. Our method is implemented as an R package called BIGRED (Bayes Inferred Genotype Replicate Error Detector), which is freely available for download: https://github.com/ac2278/BIGRED.

**Electronic supplementary material:**

The online version of this article (10.1186/s12859-018-2512-8) contains supplementary material, which is available to authorized users.

## Background

A researcher may choose, for a number of reasons, to sequence an individual multiple times, performing technical replication, biological replication, or both. Because sequencing experiments involve many steps and errors can occur during any part of the workflow, one motivation for sequencing an individual more than once is to allow researchers to compare these replicates, identify outlier samples, and evaluate how well a sequencing pipeline is executed. This is particularly important for plant breeders, as they require ongoing estimates of their program’s error rates. Further discussion of reasons for intentional replication appear elsewhere [1]. In short, the three aspects of replication—sequencing read depth, technical replication, and biological replication—each play different roles in mitigating errors that are introduced in the experimental pipeline. Increasing sequencing read depth allows for improved variant calling while technical and biological replicates allow for optimization of bioinformatic filters [1]. Replication can also arise unintentionally as a result of human error or naming inconsistencies, and it is in a researchers best interest to make full use of the data, merging the replicate records rather than discarding them.

Before merging the data from biological or technical replicates or using them to inform quality filter thresholds, it is important to verify that no erroneous samples exist among the putative replicates (i.e. verify that all putative replicates derived from an identical individual). Existing methods for error detection include performing pairwise identity-by-state and –by-descent estimation [2], calculating the correlation between pairs of samples, and examining a heat map of a realized genomic relationship matrix. These approaches require some combination of genotype calling, imputation, and haplotype phasing, making them unsuitable for low- to moderate-depth, high-throughput sequence (HTS) data [3]. And because these methods employ a pairwise-comparison approach for error detection rather than joint analysis of the samples, results may be inconsistent when more than two replicates exist. To illustrate, the general protocol for heat map analysis involves starting off with some collection of sequenced samples (including the replicates of interest), calling genotypes, filtering based on percent missing, imputing missing genotypes, calculating the additive genomic relationship matrix, and finally plotting a heat map of the putative replicates. This method can work well on deeply sequenced samples, but complications arise when applying this method to shallow-depth sequence data. Firstly, it requires genotype calling, which is difficult to do accurately when we have low read depth. Secondly, it requires imputation, raising issues in regards to reference panel and imputation method selection. Furthermore, results from imputation vary depending on which samples were jointly imputed, which in turn, affects downstream analyses that use the imputed data. Finally, a third limitation of this method—common among existing error detection methods—is that it relies on pairwise comparisons of the putative replicates, rather than joint analysis of the replicates. For example, suppose we have three putative replicates, A, B, and C. It is possible that A and B are highly correlated, A and C are highly correlated, but B and C are only moderately correlated. In situations such as this, deciding if all three samples are replicates is not straightforward.

**Fig. 1:**
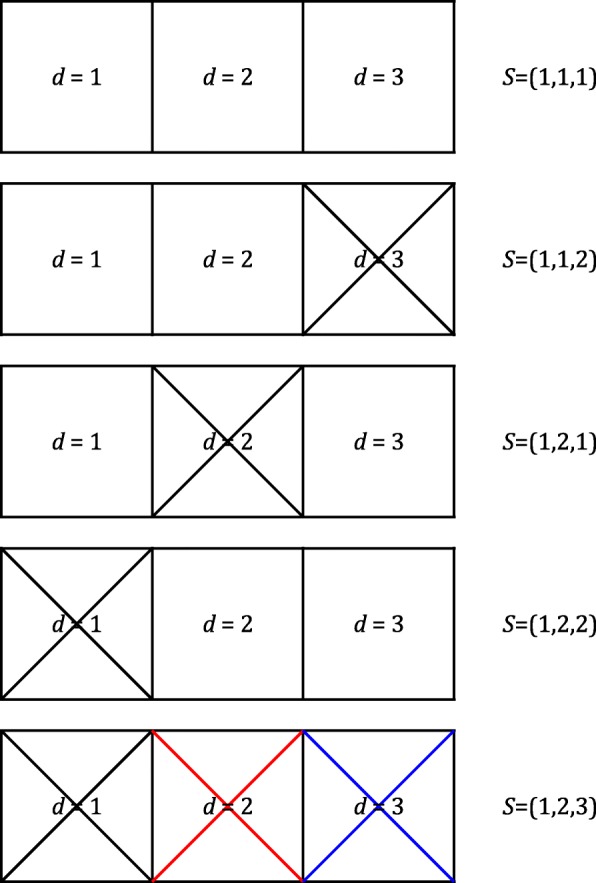
The set of relations describing the three putative replicates of an individual and the corresponding source vectors. BIGRED calculates the posterior probability distribution over the set of relations describing the putative replicates and infers which of the samples originated from an identical genotypic source. The source vector *S* = (1,2,1) represents the scenario where sample *d* = 1 and *d* = 3 originate from an identical source. Crossed out boxes represent samples without any replicate

Considering these issues, we propose a method that addresses key limitations of existing approaches. The proposed method detects errors by estimating the conditional posterior probability of all possible relationships among the putative replicates (Fig. 1). We call our algorithm BIGRED (Bayes Inferred Genotype Replicate Error Detector). BIGRED requires no genotype calling, imputation, or haplotype phasing, making it a suitable tool for studies relying on shallow-depth HTS data. We examined the effect of read depth, the number of sites analyzed (*L*), and minor allele frequency (MAF) at the *L* sites on algorithmic performance, using both real and simulated data. In this paper, we used BIGRED as a tool to verify reported replicates; however, we also envision individuals using our algorithm to test unreported but suspected replicates. Under this scheme, researchers would use some initial screening method, such as examination of the genomic relationship matrix, to identify cryptic replicates among their collection of samples and then test these suspected replicates using BIGRED.

## Methods

### The proposed method

We describe the proposed method using a case study, individual I011206 from the Next Generation (NEXTGEN) Cassava Breeding Project [4]. I011206 is recorded to have been sequenced *k* = 3 times by NEXTGEN (Additional file 1). We index the putative replicates using the variable *d*. The task is to verify that samples *d* = 1, *d* = 2, and *d* = 3 are in fact replicates of the same individual, checking all possible combinations of replicate and non-replicate status. We know that the DNA samples from these three runs can be related in one of five possible ways (Fig. 1):

1. All three samples originate from one source;
2. Samples *d* = 1 and *d* = 2 originate from one source while *d* = 3 originates from a different source;
3. Samples *d* = 1 and *d* = 3 originate from one source while *d* = 2 originates from a different source;
4. Samples *d* = 2 and *d* = 3 originate from one source while *d* = 1 originates from a different source;
5. All three samples originate from different sources.

We use “source vectors” to represent these relations and enumerate all possible source vectors for *k* = 3 on the right panel of Fig. 1. By convention: (1) source vectors are *labeled* vectors, e.g., the first, second, and third element of a given source vector describes the status of sample *d* = 1, *d* = 2, and *d* = 3, respectively, and (2) the first element of a source vector always takes on the value 1. Vector elements with the same value are indicated to be from the same source.

BIGRED detects errors by estimating the conditional posterior probability of each source vector *S*, given:

1. Estimates of population allele frequency at *L* randomly sampled biallelic sites, sampled at the genome-wide level and
2. The *k* putative replicates’ allelic depth (AD) data at the *L* sites. A site is only sampled if each putative replicate has at least one read at that site.

We make three simplifying assumptions:

1. The species is diploid;
2. Each polymorphic site harbors exactly two alleles, allele *A* and allele *B*, i.e. all polymorphisms are biallelic;
3. Sites are independent. BIGRED allows the user to specify a minimum distance, in base pairs, between any two sampled sites. The user may also filter sites based on linkage disequilibrium, although this is not a functionality of BIGRED.

### Defining a likelihood function for *G*

Let ${X}_d^{(v)}$ and ${G}_d^{(v)}$ denote the observed AD data and the underlying (unknown) genotype at site *v* for putative replicate *d*, respectively. The AD data records the observed counts of allele *A* and *B* at site *v* for sample *d*: ${X}_d^{(v)}=\left({n}_A^{\left(v,d\right)},{n}_B^{\left(v,d\right)}\right)$. Given observed data ${X}_d^{(v)}$ and fixed sequencing error rate *e*, we compute the likelihood for genotype ${G}_d^{(v)}=g$ at site *v* for sample *d* using a binomial model as follows, where *g* ∈ *AA*, *AB*, *BB*:

1 $$ {\displaystyle \begin{array}{c}P\left({X}_d^{(v)}|{G}_d^{(v)}=g,e\right)=\left(\begin{array}{c}{n}_A^{\left(v,d\right)}+{n}_B^{\left(v,d\right)}\\ {}{n}_B^{\left(v,d\right)}\ \end{array}\right){\left(1-{p}_B\right)}^{n_A^{\left(v,d\right)}}{\left({p}_B\right)}^{n_B^{\left(v,d\right)}}\\ {}{p}_B=\left\{\begin{array}{c}e,\\ {}0.50,\\ {}1-e,\end{array}\ \begin{array}{c}\mathrm{when}\\ {}\mathrm{when}\\ {}\mathrm{when}\end{array}\right.\ \begin{array}{c}g=0\kern0.5em or\  AA\\ {}g=1\kern0.5em or\  AB\\ {}g=2\kern0.5em or\  BB\end{array}\end{array}} $$

### Defining a likelihood function for *S*

**Fig. 2:**
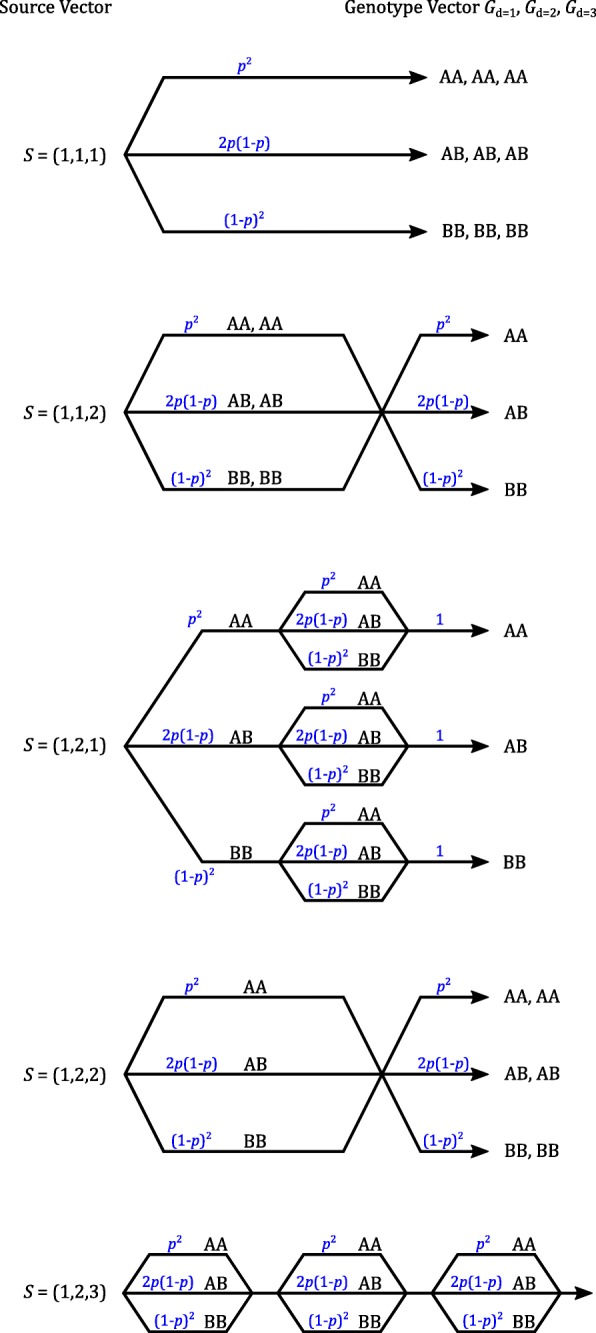
Defining *P*(*G*^(*v*)^| *S*) for *k* = 3. We first enumerate all possible source vectors of length *k* = 3 (left) then enumerate all *labeled* genotype vectors consistent with each source vector (right). Each path in a given tree corresponds to a genotype vector given source vector *S*. For instance, if the three samples are related by source vector (1,1,2), the genotype vector can take one of nine values. We compute the probability of each genotype vector (given *S*) by traversing each path and taking the product of the probabilities associated with the edges of the path. Note that genotype vectors not consistent with *S* have probability zero (we omit these paths from the figure). Edge probabilities are defined using user-supplied, population allele frequencies and assuming HWE

We walk through the procedure of defining the likelihood function for *S* when *k* = 3, continuing with individual I011206 as an example:

1. Enumerate all possible source vectors of length *k* = 3 (Fig. 1).
2. Enumerate all *labeled* genotype vectors consistent with each source vector (Fig. 2). For instance, there are three genotype vectors consistent with source vector *S* = (1,1,1): (*AA*, *AA*, *AA*), (*AB*, *AB*, *AB*), and (*BB*, *BB*, *BB*). There are nine genotype vectors consistent with *S* = (1,1,2): (*AA*, *AA*, *AA*), (*AA*, *AA*, *AB*), (*AA*, *AA*, *BB*), (*AB*, *AB*, *AB*), (*AB*, *AB*, *AA*), (*AB*, *AB*, *BB*), (*BB*, *BB*, *BB*), (*BB*, *BB*, *AA*), and (*BB*, *BB*, *AB*).
3. Define a likelihood function for *S* as a function of genotype likelihoods, defined previously in Eq. 1:

2 $$ {\displaystyle \begin{array}{c}P\left({X}^{(v)}|S\right)=\sum \limits_{G^{(v)}}P\left({X}^{(v)},{G}^{(v)}|S\right)\\ {}=\sum \limits_{G^{(v)}}P\left({X}^{(v)}|{G}^{(v)}\right)P\left({G}^{(v)}|S\right)\\ {}=\sum \limits_{G^{(v)}}\left[\prod \limits_{d=1}^kP\left({X}_d^{(v)}|{G}_d^{(v)}\right)\right]P\left({G}^{(v)}|S\right)\end{array}} $$

The function *P*(*G*^(*v*)^| *S*) is the probability that the *k* samples have genotype vector ${G}^{(v)}=\left({G}_{d=1}^{(v)},{G}_{d=2}^{(v)},\dots, {G}_{d=k}^{(v)}\right)$given that source vector *S* describes how the *k* samples are related. We define *P*(*G*^(*v*)^| *S*) using the (user-supplied) population allele frequency of allele *B* at site *v* and assuming Hardy-Weinberg Equilibrium (HWE; Fig. 2). For samples that are encoded as identical in source vector *S*, we treat their genotypes as a single observation and all non-identical genotypes are modeled as independent (Fig. 2).

### Estimating *P*(*S*| *X*)

Once we compute *P*(*X*^(*v*)^| *S*) at all *L* sites, we compute *P*(*S*| *X*) jointly across all *L* sites using Eq. 3 and assuming a uniform prior on *S*:

3 $$ {\displaystyle \begin{array}{c}\prod \limits_{v=1}^L\kern0.5em P\left({X}^{(v)}|S\right)=P\left(X|S\right)\\ {}P\left(S|X\right)=\frac{P\left(X|S\right)P(S)}{\sum_S\left[P\left(X|S\right)P(S)\right]}\end{array}} $$

One may wish to compare the posterior probability of two assignments of *S*, and when doing so via the posterior odds-ratio, both the denominator and *P*(*S*) cancel from the two posteriors (since the denominator acts as a normalizing constant and we assume a uniform prior on *S*). The ratios of the posteriors are, therefore, equal to the ratios of the likelihoods.

### Evaluating BIGRED

We examined how changes in mean read depth, *L*, and MAF at the *L* sites affect the accuracy of BIGRED. For simulation experiments, we used a fixed sequencing error rate of 0.01 and sampled sites such that no two sites fell within 20 kb from one another. In addition to accuracy, we evaluated the sensitivity of the algorithm. We used high-depth whole-genome sequence (WGS) data from 241 *Manihot esculenta* individuals to simulate a series of data sets. Filtering the data (e.g., removing sites with extremely low minor allele frequency and discarding regions prone to erroneous mapping) should be done prior to applying BIGRED to remove potentially spurious variants. We refer the reader to the section “Alignment of reads and variant calling of cassava haplotype map (HapMapII)” of [5] for a description of how the data was generated and the quality filters applied.

### The data

**Fig. 3:**
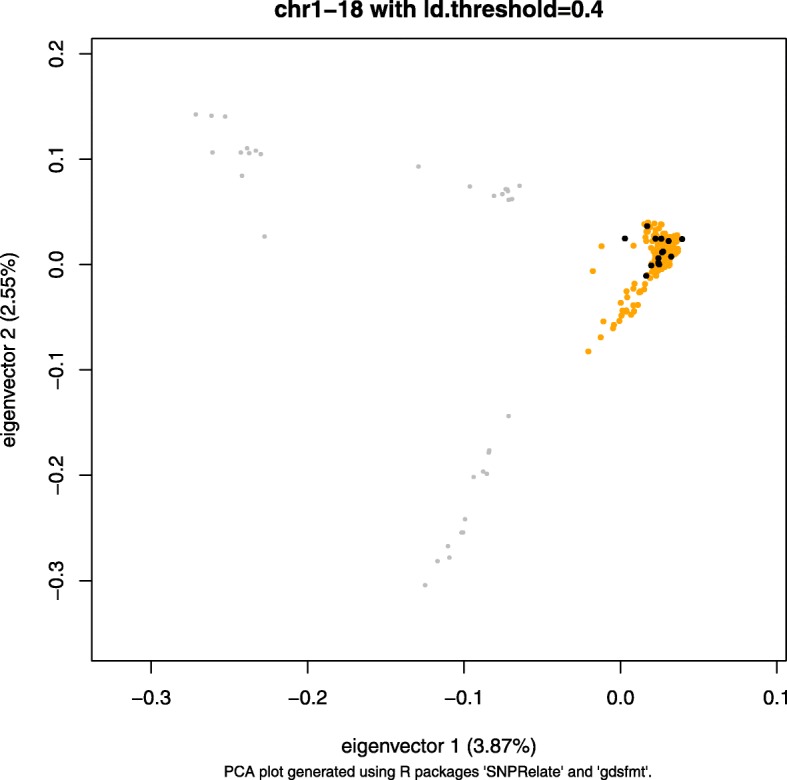
PCA on 241 *Manihot esculenta* genotypes, using a subset of SNPs in approximate linkage equilibrium. The x-axis and y-axis in this figure represents the first and second eigenvector, respectively. The 241 individuals clustered into roughly three groups. We used cultivated cassava (orange and black) to evaluate BIGRED in simulation experiments. We used 15 individuals (black) to simulate AD data and all 206 (orange and black) individuals to estimate population allele frequencies at sites

The WGS data consist of both AD data and called genotypes for 241 individuals. To detect the presence of any population structure, we performed principal component analysis (PCA) using the called genotypes for the 241 individuals. We generated a pruned subset of SNPs that are in approximate linkage equilibrium with each other and then performed a PCA using this pruned subset of SNPs (Fig. 3). We performed LD-based SNP pruning and PCA using R packages SNPRelate() and gdsfmt() with a LD threshold of 0.40 [6]. The 241 individuals clustered into roughly three groups. The 206 individuals shown in orange represent cultivated cassava. We used these 206 individuals to estimate population allele frequencies at sites and 15 individuals, previously found to be genetically distinct [7], to simulate AD data for experiments. We limited our simulation experiments to these 15 members to ensure that all individuals truly represent distinct genotypes rather than only nominally distinct.

### Simulation experiments to evaluate the impact of mean read depth and MAF on accuracy

We first evaluated the effect of mean read depth *λ* and MAF on the algorithm’s accuracy, holding *L* constant at 1000 sites. We outline the procedure to simulate AD data for the scenario where *k* = 3 and *S =* (1,2,1):

1. Enumerate all possible pairs of genotypes, where order does not matter (*n* = 15(14) = 210).
2. Sample one genotype pair.
3. Randomly assign the status ‘source 1’ to one of the two genotypes. Assign the remaining genotype ‘source 2’ status.
4. Randomly sample *L* = 1000 sites (genome-level) with a specified MAF.
5. Simulating ${X}_{d=1}^{(v)}$: Sample *Y* alleles (with replacement) from the pool of allele reads belonging to source 1 at that site, where *Y*~*Poisson*(*λ*).
6. Simulating ${X}_{d=2}^{(v)}$: Sample *Y* alleles (with replacement) from the pool of allele reads belonging to source 2 at that site, where *Y*~*Poisson*(*λ*).
7. Simulating ${X}_{d=3}^{(v)}$: Sample *Y* alleles (with replacement) from the pool of allele reads belonging to source 1 at that site, where *Y*~*Poisson*(*λ*).
8. Feed the algorithm the simulated AD data and the population allele frequency of allele *B* at the *L* sites.
9. Record the conditional posterior probability of *S =* (1,2,1).
10. Repeat steps 2 through 9, 100 times. When repeating step 2, only sample from those genotype pairs that have not been sampled previously.

Note that evaluating scenario *S =* (1,2,1) is equivalent to evaluating scenarios *S =* (1,1,2) and *S =* (1,2,2). We performed a full factorial experiment for the source vectors associated with *k* = 2, *k* = 3, and *k* = 4, where *λ* = {1,2,3,6,15} and where we sampled sites with a given MAF falling in one of five possible intervals (0.0,0.1], (0.1,0.2], (0.2,0.3], (0.3,0.4], and (0.4,0.5]. Note that in these simulation experiments, all putative replicates of a given individual had identical mean read depths. We later tested the scenario where mean read depths varied among the samples.

### Simulation experiments to evaluate the impact of *L* on accuracy

To assess the impact of *L* on accuracy, we repeated simulation experiments for *S =* (1,2,1) and *S =* (1,2,3), sampling sites with MAFs falling in (0.2,0.3] and testing seven values of *L*: 50, 100, 250, 500, 1000, 2000, and 5000.

### Simulation experiments to evaluate BIGRED’s sensitivity

We next evaluated the algorithm’s sensitivity by simulating the scenario where *S =* (1,1) and corrupting (i.e., contaminating) *p* percent of sites in sample *d* = 2 with a second, randomly sampled genotype source. We tested five values of *p* (10, 20, 30, 40, 50%) at five mean depths (1x, 2x, 3x, 6x, and 15x). We repeated this procedure 100 times for each depth and *p* combination.

### Simulation experiments to evaluate the scenario where mean read depths vary among the *k* putative replicates

We simulated data for three source vectors *S =* (1,1), *S =* (1,2), and *S =* (1,2,1). For *S =* (1,1) and *S =* (1,2), we varied the mean read depth of sample *d* = 2 while keeping the mean depth of sample *d* = 1 constant at 1x. We tested five different *λ* values for sample *d* = 2: 1, 2, 4, 6, and 12. For *S =* (1,2,1), we varied the mean read depth of sample *d* = 3 while keeping the mean depth of samples *d* = 1 and *d* = 2 constant at 1x. We again tested five *λ* values for sample *d* = 2: 1, 2, 4, 6, and 12. We held *L* constant at 1000 across all experiments and tested the same five MAF intervals as before.

#### Comparing results to hierarchical clustering

**Fig. 4:**
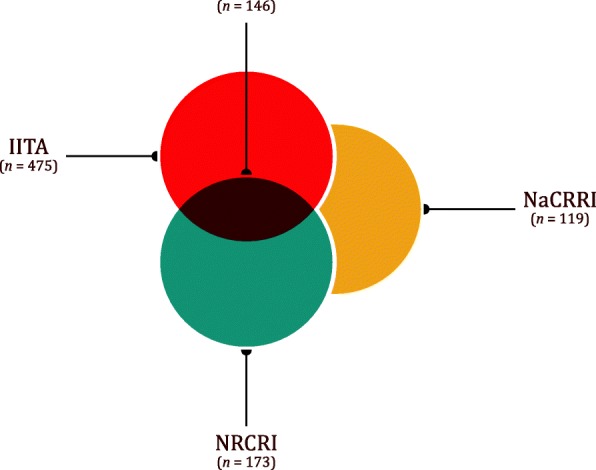
A Euler diagram showing the number of cases (*n*) where a given genotype has been sequenced more than once. We found *n* = 475 genotypes (excluding TMEB419) within the IITA germplasm collection that have each been sequenced *k* > 1 times. Entries falling at the intersection of IITA and NRCRI (black) represent cases where IITA submitted DNA for *k*-*x* sequence runs of a given genotype and NRCRI submitted DNA for the remaining *x* runs. There were 146 such cases. We found *n* = 173 genotypes within the NRCRI germplasm collection that have each been sequenced *k* > 1 times. We found *n* = 119 genotypes within the NaCRRI germplasm collection that have each been sequenced *k* > 1 times

To compare results from BIGRED and hierarchical clustering, we used genotyping-by-sequencing (GBS) data [8] collected by three of the four breeding programs collaborating on the NEXTGEN Project: the International Institute of Tropical Agriculture (IITA), the National Crops Resources Research Institute (NaCRRI), and the National Root Crops Research Institute (NRCRI). We refer the reader to the section “Data generation and variant calling” of [9] for a description of how the data were generated and filtered. We estimated non-replicate rates for these three programs. Additional files 2, 3, and 4 list the names of the *k* putative replicates associated with a given genotype from IITA, NaCRRI, and NRCRI, respectively. The Euler diagram below shows the number of cases where a given genotype has *k* > 1 sequence records, for each breeding institution (Fig. 4). We found *k* = 9 samples associated with TMEB419, a genotype used in breeding efforts at both IITA and NRCRI, and excluded this genotype from our analysis due to the computational demands for cases where *k* > 7. Additional file 5 plots the number of source vectors associated with *k* for *k* ∈ {1, …, 8}. We also removed putative replicates with a genome-wide mean read depth below 0.5 (Additional file 6 lists the samples removed). We ran BIGRED using *L* = 1000 randomly sampled sites across cassava’s 18 chromosomes with MAFs falling between (0.4,0.5]. No two sites fell within 20 kb from one another, and we assumed a fixed sequencing error rate of 0.01 when calculating genotype likelihoods.

We compared results from BIGRED to results obtained from hierarchical cluster analysis. Results from [10] show that hierarchical clustering is an effective tool for matching accessions from farmers’ fields to corresponding varieties in an existing database of known varieties, a problem very similar to the one being addressed in this paper. We performed hierarchical clustering on the *k* putative replicates of each genotype. To do this, we first calculated the realized additive relationship matrix for the 1215 sequenced samples from IITA using sites harboring biallelic SNPs. Sites were filtered using criteria based on MAF and percent missing. Sites with a MAF falling within the interval (0.1,0.5] and with < 50% missing data across the 1215 samples were kept, leaving us with 46,862 sites (out of 100,267) to analyze. We calculated the realized additive relationship matrix using the A.mat() function from the R package rrBLUP [11]. We used a matrix of genotype dosages as input and imputed missing dosage values using the “mean” option. We then calculated a distance matrix between the rows of the additive relationship matrix using Euclidean distance as the distance measure. We performed complete-linkage hierarchical clustering using the hclust() function and the distance matrix as input [12]. For each genotype, the hclust() function returns a tree structure with *k* leaves, each leaf representing a putative replicate. We determined the underlying relationship among each genotype’s putative replicates by cutting each tree at a height of 0.5. We refer to this relationship as the “source vector” to keep terminology consistent with that of BIGRED’s. We compared results from the complete-linkage cluster analysis to results from BIGRED. For BIGRED, we set a posterior probability threshold of 0.99, i.e., BIGRED would only return an inferred source vector if that source vector had a posterior probability of at least 0.99. This minimum posterior probability threshold was met in all cases, i.e., we were able to infer a source vector in all cases. We repeated this procedure for NaCRRI (299 sequenced samples and 48,712 sites) and NRCRI (415 sequenced samples and 48,320 sites).

For each breeding institution, we categorized the institution’s genotypes into groups based on the number of putative replicates (*k*) each genotype had. We then calculated a mean non-replicate rate μ_k_ separately for each *k*. To calculate this, we computed a non-replicate rate for each individual that has *k* putative replicates (when *k* = 2, this rate is 1 - *P*(*S* = (1,1)|*X*)), and then averaged these values across all individuals of a given *k*.

#### Comparing the consistency of BIGRED and hierarchical clustering

To compare the consistency of BIGRED and hierarchical clustering, we performed a set of experiments using the GBS data from the 475 IITA individuals with 1 < *k* < 7 putative replicates. The basic premise of these experiments is that an analysis based on a larger set of sites is likely to be correct. The first step in these experiments is to perform error detection on an individual’s putative replicates using the data at a large number of sites and to set the inferred source vector as the “truth”. The second step is to perform error detection once more on the individual’s replicates, this time using the data at a smaller number of sites disjoint from the initial set. To obtain a measure of consistency, we compare the results from the first (larger) analysis with results from the second (smaller) analysis.

To evaluate the consistency of hierarchical clustering, we first filtered the data, retaining samples with a genome-wide mean read depth of ≥0.5 and sites with MAFs within the interval (0.3,0.5] and with < 50% missing data across the filtered samples. This left 1215 samples and 16,926 sites for analysis. As before, we called genotype dosages using the observed allelic read depth data and imputed missing values at a given site with the site mean. We then performed hierarchical clustering on each of the 475 individuals, using data from 2000 randomly sampled sites. We set the output of these analyses as the “truth”. We then performed hierarchical clustering on each of the individuals a second time, sampling *L* sites disjoint from the initial 2000, and compared the inferred source vector with the “true” source vector. We tested five values of *L*: 50, 100, 250, 500, and 1000. We repeated the experiment 10 times for each value of *L* and calculated a mean concordance rate between the “true” source vector and the source vector inferred from the *L* sites across the 10 runs and 475 cases for each *L*.

To evaluate the consistency of BIGRED, we first filtered the data, keeping samples with a genome-wide mean read depth of ≥0.5 and sites with MAFs within the interval (0.3,0.5]. As with hierarchical clustering, we defined the truth using 2000 randomly sampled sites. We used a fixed sequencing error rate of 0.01 and sampled sites such that no two sites fell within 20 kb from one another. We followed the same procedure as the one used to evaluate the consistency of hierarchical clustering, in particular, testing with the same five values of *L*.

### Applying a pairwise-comparison approach to real data

Methods that employ a pairwise-comparison approach for error detection rather than joint analysis of the samples might produce ambiguous results when more than two putative replicates exist. To demonstrate, we applied a pairwise-comparison method to IITA’s data, specifically we calculated the Pearson correlation between all pairs of putative replicates. We refer to this method as the “correlation method”. Before calculating the Pearson correlation between replicate pairs, we filtered the data, retaining samples with a genome-wide mean read depth of ≥0.5, sites with MAFs within the interval (0.3,0.5], and with < 50% missing data across the filtered samples. This left 1215 samples and 16,926 sites for analysis. We called genotype dosages using the observed allelic read depth data and imputed missing values using glmnet [9]. We then calculated the Pearson correlation between all pairs of putative replicates using the cor() function [12]. For simplicity, we limited our analysis to the 154 cases where *k* = 3. Correlations ranged from 0.02 to 0.93, so we selected 0.85 as the replicate-call threshold (i.e., two putative replicates with a correlation ≥0.85 are considered true replicates). We also applied a replicate-call threshold of 0.80 to examine how results changed.

### Run time

We measured computation time as the number of central processing unit (CPU) seconds required to run BIGRED. All jobs were submitted to the Computational Biology Service Unit at Cornell University, which uses a 112 core Linux (CentOS 7.4) RB HPC/SM Xeon E7 4800 2 U with 512GB RAM.

## Results

### Evaluating the accuracy and run-time of BIGRED

**Fig. 5:**
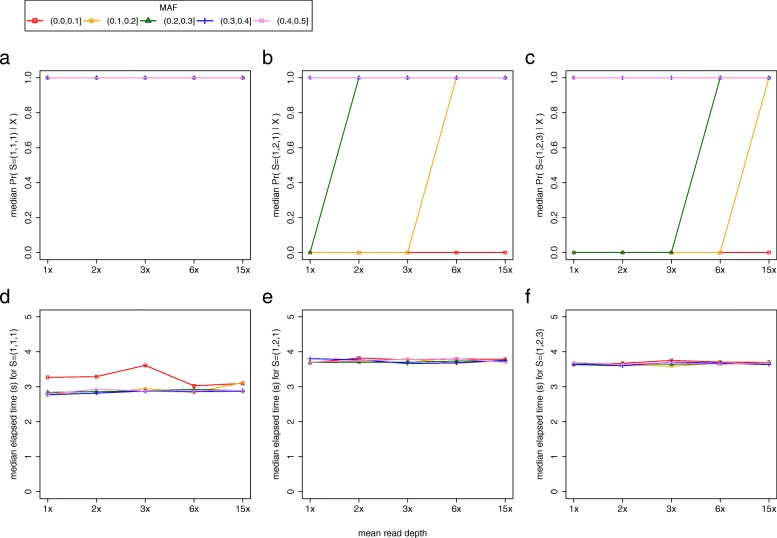
Algorithm’s accuracy and run-time as a function of the mean read depth of samples and the MAF of analyzed sites for *k* = 3. (**a**, **b**, and **c**) Each plot shows estimates of the median posterior probability of the true source vector (*y*-axis) as a function of mean read depth of samples (*x*-axis) and MAF of sites (legend). Each data point presents the median posterior probability of *S =* (1,1,1) across 15 runs, *S =* (1,2,1) across 100 runs, and *S =* (1,2,3) across 100 runs of the algorithm. (**d**, **e**, and **f**) Each plot shows the mean elapsed time in seconds for each simulation scenario

To evaluate the algorithm’s accuracy and run-time, we performed a full factorial experiment where we simulated data for each of the source vectors associated with *k* = 2, 3, and 4, varying the mean read depth of samples and the MAF of the *L* = 1000 sites sampled by the algorithm. We use the term “accuracy” to refer to the median posterior probability of the true source vector. For these experiments, we simulated the situation where all *k* putative replicates had identical mean read depths but later tested the scenario where mean read depths varied among the *k* samples (refer to the section “Evaluating BIGRED’s accuracy when mean read depths vary among the *k* putative replicates”). We observed qualitatively similar results for *k* = 2, 3, and 4, so we present only the results for *k* = 3 in the main text (Fig. 5). We present the results for *k* = 2 and 4 in Additional file 7. When no erroneous samples were present among the *k* putative replicates, the algorithm performed consistently well across all mean read depths and MAF intervals, assigning a median posterior probability of one to the true source vector (Fig. 5a). We observed a common trend for the remaining two source vectors: for a given MAF interval, accuracy monotonically increased as mean read depth increased. We observed this trend in all cases except for interval (0.0,0.1], whose median accuracy stayed constant at zero across all depths for *S =* (1,2,1) and *S =* (1,2,3) and intervals (0.3,0.4] and (0.4,0.5], whose median accuracies stayed constant at one across all depths for *S =* (1,2,1) and *S =* (1,2,3) (Fig. 5b and c). In addition to recording the posterior probability of the true (simulated) source vector, we also recorded the posterior probability assigned to all other source vectors. We present the plots for *S =* (1,2,1) and *S =* (1,2,3) experiments in Additional file 8. These plots recapitulate the behavior observed in Fig. 5 but do so at a higher resolution: for a given MAF interval, with the exception of (0.0,0.1], BIGRED shifts the probability away from *S* = (1,1,1) towards the true (simulated) source vector as the mean read depth of samples increases. The algorithm takes, on average, approximately three seconds to analyze all possible source vectors when the true source vector is *S =* (1,1,1) for all pairwise combinations of sample mean read depth and site MAF interval (Fig. 5d). Similarly, the algorithm takes, on average, approximately four seconds to analyze all possible source vectors when the true source vectors were *S =* (1,2,1) and *S =* (1,2,3) for all pairwise combinations of sample mean read depth and site MAF interval (Fig. 5e and f).

**Fig. 6:**
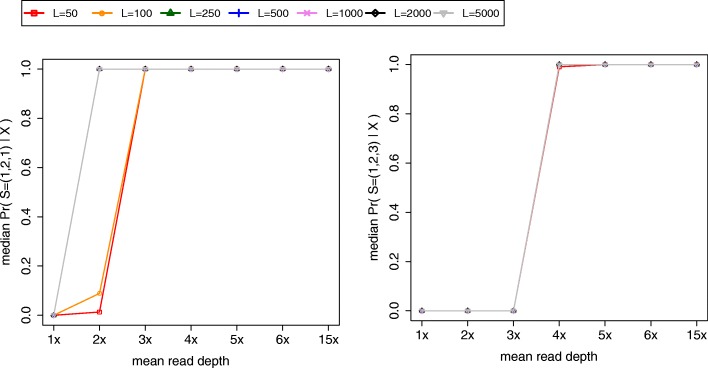
The impact of *L* on accuracy. The two plots show estimates of the median posterior probability of the true source vector (*y*-axis) as a function of mean read depth of samples (*x*-axis) for different values of *L* (legend). We sampled sites whose MAFs fell in the interval (0.2,0.3]

To assess the impact of *L* on the algorithm’s accuracy, we repeated simulation experiments for *S =* (1,2,1) and *S =* (1,2,3), this time varying values of *L* and looking only at sites with MAFs falling in (0.2,0.3]. We tested the (0.2,0.3] interval since median accuracy was one for all earlier experiments using intervals (0.3,0.4] and (0.4,0.5]. We tested seven values of *L*: 50, 100, 250, 500, 1000, 2000, and 5000. Median accuracy drastically increased when *L* increased from 100 to 250 for *S* = (1,2,1) at 2x mean depth (Fig. 6a). At a given mean read depth, we observed little to no change in median accuracy when increasing *L* for *S* = (1,2,3) (Fig. 6b).

### Evaluating the sensitivity of the algorithm

**Fig. 7:**
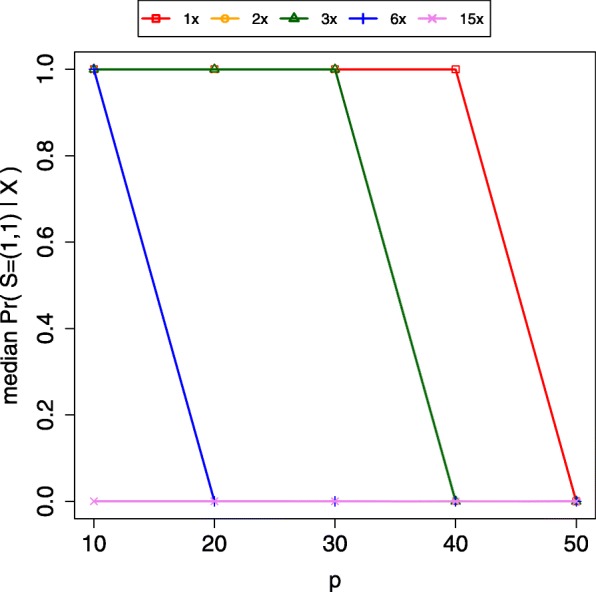
Algorithm’s sensitivity as a function of the mean read depth of samples. We assessed the impact of mean read depth on the method’s sensitivity. The plot reports estimates of the median posterior probability of the true source vector *S =* (1,1) (*y*-axis) as a function of the percentage of contaminated sites (*p*) in sample *d* = 2 (*x*-axis) and mean read depth of putative replicates (legend). In these experiments, samples *d* = 1 and *d* = 2 have identical mean read depths

To evaluate the algorithm’s sensitivity, we first simulated the scenario where *S =* (1,1) then contaminated *p* percent of sites in sample *d* = 2 with a second genotypic source. We then assessed how much probability the algorithm assigned to source vector *S =* (1,1) in light of these contaminated sites. We tested five different values of *p* in combination with five sample mean read depths. The algorithm showed greater sensitivity to increases in *p* as the mean read depth of the samples increased (Fig. 7).

### Evaluating BIGRED’s accuracy when mean read depths vary among the *k* putative replicates

**Fig. 8:**
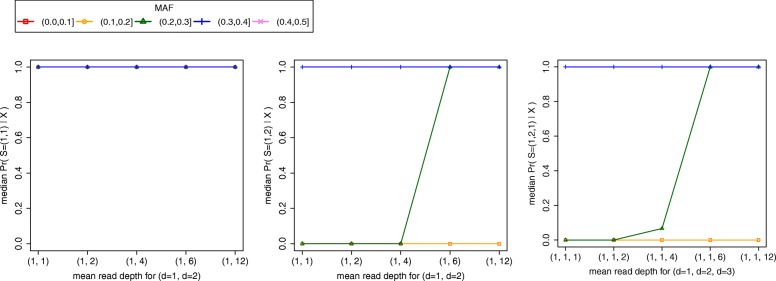
Accuracy of the algorithm when the mean read depths of the *k* putative replicates vary. Each data point in the three plots reports the median posterior probability for the true source vector (y-axis) as a function of the mean read depth for the *k* samples (*x*-axis) and the MAF of sampled sites (legend)

We next evaluated the algorithm’s accuracy when the read depths vary among the *k* samples. For these experiments, we examined three source vectors *S =* (1,1), *S =* (1,2), and *S =* (1,2,1) and used *L* = 1000 sites. And as before, we examined the impact of MAF at the 1000 sites. When simulating data for source vectors *S =* (1,1) and *S =* (1,2), we varied the mean read depth of sample *d* = 2 while keeping the mean depth of sample *d* = 1 constant at 1x. We tested five different read depth values for sample *d* = 2 (*λ* = 1, 2, 4, 6, and 12). When simulating data for source vector *S =* (1,2,1), we varied the mean read depth of sample *d* = 3 while keeping the mean depth of samples *d* = 1 and *d* = 2 constant at 1x. We tested five different read depth values for sample *d* = 3 (*λ* = 1, 2, 4, 6, and 12). We obtained results comparable to those from simulation experiments where all *k* putative replicates had identical mean read depths. For *S =* (1,1), the algorithm performed consistently well across all read depth differences and MAF intervals, assigning a median posterior probability of one to the true source vector (Fig. 8a). For *S =* (1,2) and *S =* (1,2,1), the algorithm performed consistently well across all read depth differences when analyzing sites with MAFs falling in (0.3,0.5] and consistently poorly across all read depth differences when analyzing sites with MAFs falling in (0.0,0.2] (Fig. 8b and c). For MAF interval (0.2,0.3], median accuracy monotonically increased as the difference between sample read depths grew, i.e. as the mean read depth for sample *d* = 2 in *S =* (1,2) and *d* = 3 in *S =* (1,2,1) increased (Fig. 8b and c).

### Estimating NEXTGEN non-replicate rates

We estimated non-replicate rates μ_k_ for IITA, NaCRRI, NRCRI, and the germplasm used by both IITA and NRCRI, respectively (Table 1).

**Table 1:** A table summarizing the mean non-replicate rate μ_k_ of each breeding institution

	Institution	*k* = 2	*k* = 3	*k* = 4	*k* = 5	*k* = 6
*n*_*k*_	IITA	272	154	37	11	1
*μ*_*k*_	IITA	0.21	0.16	0.14	0.27	1
*n*_*k*_	NaCRRI	58	61	0	0	0
*μ*_*k*_	NaCRRI	0.05	0.21	–	–	–
*n*_*k*_	NRCRI	128	31	5	8	1
*μ*_*k*_	NRCRI	0.37	0.32	0.40	0.25	1
*n*_*k*_	IITA & NRCRI	101	31	5	8	1
*μ*_*k*_	IITA & NRCRI	0.33	0.32	0.40	0.25	1

For each institution, we categorized genotypes into groups based on the number of putative replicates each genotype had. Grey rows show the number of genotypes in each group *n*_*k*_ for each breeding institution. We then calculated the mean non-replicate rate among genotypes of a given *k μ*_*k*_ by calculating the mean probably of no errors then subtracting this value from one.

### Method comparison

**Fig. 9:**
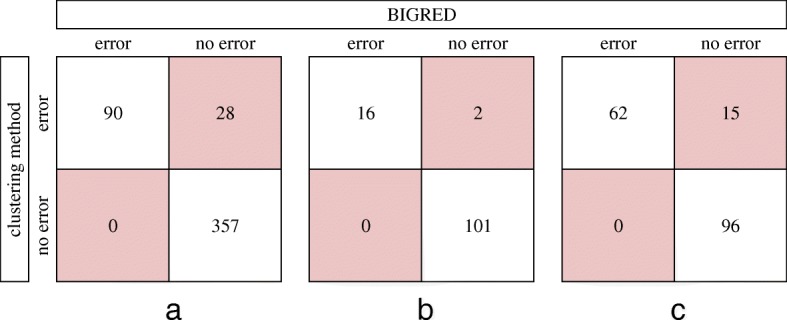
Comparing results from complete-linkage hierarchical clustering and the proposed method. Above are three two-way contingency tables comparing the results from complete-linkage hierarchical cluster analysis and the proposed method for IITA (**a**), NaCRRI (**b**), and NRCRI (**c**). Conflicts between the two methods are shown in red. The 146 genotypes shared between IITA and NRCRI (Fig. 4; black) are represented twice in our results: once with the 329 genotypes unique to IITA and once with the 27 genotypes unique to NRCRI

We compared results from BIGRED to results obtained from complete-linkage hierarchical cluster analysis. The two methods reported 28, 2, and 15 conflicting results for IITA, NaCRRI, and NRCRI, respectively (Fig. 9), all of which were cases where hierarchical clustering reported an error among putative replicates while BIGRED reported no error, with the exception of one NRCRI individual UG120041. Both methods reported an error for UG120041 but reported different errors: BIGRED inferred a (1,2,3) relationship while hierarchical clustering inferred a (1,1,2) relationship.

We compared the consistency of BIGRED with that of hierarchical clustering. Table 2 presents the mean concordance rate between the “true” source vector and the source vector inferred from *L* sites among 475 cases across the 10 runs of hierarchical clustering and BIGRED. BIGRED had a higher concordance rate than hierarchical clustering at every *L*, suggesting that BIGRED is a more consistent estimator than hierarchical clustering.

**Table 2:** A table comparing the consistency of BIGRED and hierarchical clustering using the 475 IITA individuals with 1 < *k* < 7 putative replicates

Method	*L* = 50	*L* = 100	*L* = 250	*L* = 500	*L* = 1000
BIGRED	0.9832	0.9895	0.9958	0.9973	0.9981
Hierarchical clustering	0.8322	0.9088	0.9488	0.9640	0.9771

To evaluate the consistency of the two methods, we performed error detection on an individual’s putative replicates using the data at 2000 sites and set the inferred source vector as the “truth”. We then performed error detection a second time using a smaller number of sites (*L*) disjoint from the initial set. We compared the “true” source vector with the source vector inferred from *L* sites. For each IITA individual, we tested five values of *L* and repeated the experiment 10 times for each value of *L*. We then calculated the mean concordance rate between the “true” source vector and the source vector inferred from *L* sites across the 475 cases and across 10 runs.

One motivation for BIGRED’s joint analysis framework is that pairwise-comparison methods might produce ambiguous results for cases of more than two putative replicates. We introduced a hypothetical example of this in the Background section and found real examples of these inconsistencies when applying a pairwise-comparison method to IITA’s data. More specifically, when examining cases of *k* = 3 and using a replicate-call threshold of 0.85, we found 80 cases (out of 154) where the pairwise method awarded any pair of samples (of an individual) replicate status. Of these 80 cases, we found 10 cases where the method produced ambiguous results (Additional file 9). When we decreased the call threshold to 0.80, we found 146 cases where the method inferred at least one true replicate pair but six of these cases had ambiguous results (Additional file 10).

## Discussion

Researchers may choose, for a number of reasons, to sequence a given individual more than once. Regardless of intent, it is important to identify potentially mislabeled or contaminated samples before using the data (e.g. merging the data from replicate sequence runs or using the data to optimize bioinformatics quality filters). Unfortunately, existing methods to detect such errors are ad hoc and ill suited for use in shallow-depth HTS data since they require some combination of genotype calling, imputation, and haplotype phasing. We have introduced a new probabilistic framework for error detection that addresses key limitations of existing methods. Using Bayes Theorem, we calculate the posterior probability distribution over the set of relations describing the putative replicates (i.e. the set of source vectors), allowing us to infer which of the samples originated from an identical genotypic source.

We examined the impact of mean read depth, *L*, and MAF at the *L* sites on the accuracy of the proposed method through a series of simulation experiments. We found that the algorithm is most accurate when analyzing sites whose MAFs fall in the range (0.3,0.5], consistently across all mean read depths when *L* = 1000 (Fig. 5). Sites with MAFs falling in the interval (0.0,0.1] relay little information to the algorithm. When analyzing these sites, BIGRED assigns a median posterior probability of one to *S* = (1,1,1), regardless of the true source vector. Thus BIGRED appears to be biased towards inferring no error among putative replicates when analyzing sites with low MAF. One reason for this bias is our definition of *P*(*G*^(*v*)^|*S*) (Fig. 2). Given a site that has a reference allele frequency of 0.1, when *k* = 3, the probability of *G*^(*v*)^ = (AA,AA,AA) given *S* = (1,1,1), i.e. no erroneous samples among the putative replicates, is 0.1^2^, whereas the probability of *G*^(*v*)^ = (AA,AA,AA) given any other source vector is ≤0.1^4^. This bias is compounded by the fact that we estimated allele frequencies from a set of 206 individuals but ran simulation experiments using a subset of 15. Some loci that had low but non-zero MAF among the 206 individuals appeared monomorphic among the 15 individuals, making the 15 individuals look more similar than they actually are in reality. We found that 47.14 and 5.29% of sites with MAFs in the (0.0,0.1] and (0.1,0.2] interval, respectively, became monomorphic among the 15 individuals.

To evaluate the impact of *L* on the algorithm’s accuracy, we repeated simulation experiments for *S* = (1,2,1) and *S* = (1,2,3) using different values of *L* and looking only at sites with MAFs falling in (0.2,0.3]. Surprisingly, we observed little to no change in median accuracy at a given depth when increasing the number of sampled sites. The only exception was *S* = (1,2,1) at 2x mean depth, where we observed a drastic increase in accuracy when increasing *L* from 100 to 250 (Fig. 6). For *S* = (1,2,3) at 2x and 3x, we observed a median accuracy of zero even when sampling 5000 sites. We observed an increase in median accuracy only after increasing the mean read depth of samples to 4x. These results indicate that the mean read depth of samples contributes more to accuracy than the number of sampled sites. In these simulation experiments, all *k* putative replicates of a given genotype were assigned identical mean read depths. These results, however, were robust to samples with varying mean read depths (Fig. 8).

We also assessed the sensitivity of the algorithm as a way to gauge how the proportion of exogenous DNA affects the algorithm and how allelic sampling bias impacts results. The GBS protocol uses methylation-sensitive restriction enzymes (REs) to avoid sampling highly repetitive regions of the genome. One potential complication when using methylation-sensitive REs is allelic sampling bias of a marker or unequal sampling and sequencing of homologous chromosomes, resulting from differential methylation in a region. ApeKI, the RE employed by NEXTGEN, for instance, will not cut if the 3′ base of the recognition sequence on both strands is 5-methylcytosine. To test the impact of imperfect marker “heritability”, we simulated the scenario where *S* = (1,1) and corrupted *p* percent of sites in sample *d* = 2 with a second genotype source. We tested the cases where *p* = {10,20,30,40,50%} for five different sample mean depths (*λ* = {1,2,3,6,15}) and found that the algorithm was robust to increases in *p* for lower values of *λ* (Fig. 7). Not surprisingly, the method assigned higher probability to *S* = (1,2) as *p* and mean depth increased. As mean depth increases, the algorithm grows increasingly confident that differences at sites reflect true biological differences rather than sampling variation or error.

When applying BIGRED and hierarchical clustering on real data, we found a relatively high concordance rate between the two methods (Fig. 9). Although this comparison does not directly tell the reader which of the two methods is more accurate, the comparison and the analyses in this paper demonstrate the benefits of using BIGRED over hierarchical clustering. Firstly, we found that BIGRED is a more consistent estimator relative to hierarchical clustering (Table 2). Secondly, BIGRED employs a probabilistic framework to tackle the problem of error detection rather than a heuristic one like hierarchical clustering, making BIGRED a more statistically rigorous and neatly packaged method. Hierarchical clustering requires the user to make many (arguably arbitrary) decisions throughout the protocol, whereas BIGRED requires the user to make one decision at the very end, i.e. the probability at which to “call” a source vector. Our results also highlight one of the major flaws of methods like hierarchical clustering: results can change depending on what samples were included in the analysis, specifically during imputation. There are 146 genotypes that are used in both IITA’s and NRCRI’s breeding programs, and these 146 genotypes appear in both institutions’ data (Fig. 4). We performed hierarchical clustering on these individuals a total of two different times: once in combination with the 329 genotypes unique to IITA and once in combination with the 27 genotypes unique to NRCRI. Ideally, the duplicate runs of an individual would produce identical results, regardless of what other samples where included in each analysis. Of the 146 cases, however, we found three cases where the hierarchical clustering-based duplicate analyses produced conflicting results: one case where the two analyses reported differ errors and two cases where the IITA analysis reported no error but the NRCRI analysis reported an error. These conflicts likely resulted from the imputation component of the cluster analysis procedure since sample composition is known to affect imputation. These issues highlight the benefits of our approach: when we ran BIGRED on these 146 individuals twice, we found that all duplicate runs produced identical results.

In our simulation experiments, we estimated allele frequencies from WGS data. Users of BIGRED will likely not have this option and will need to estimate allele frequencies using low- to moderate-depth sequence data. Although such frequency estimates will in general contain noise, we showed that BIGRED is robust to imperfect estimates of allele frequency. We estimated allele frequencies from a set of 206 individuals but ran simulation experiments using a subset of 15 individuals and were able to recover the true underlying source vector when analyzing sites with MAFs falling in the (0.3,0.5] interval (Fig. 5). We also suggest that a user perform preliminary analyses (e.g., with PCA) to detect the presence of population structure, and when structure is evident, we recommend analyzing subpopulations separately, estimating allele frequencies from samples of a given subpopulation then running BIGRED on the samples from that subpopulation.

The number of possible source vectors increases exponentially as *k* increases (Additional file 5). For this reason, we do not recommend using BIGRED on cases where *k* > 7. We, however, do not anticipate many scenarios where a researcher would have sequenced a given individual more than seven times, but if this scenario does occur, one could either randomly select seven putative replicates to analyze or divide the replicates into sets of no more than seven samples. If using the latter scheme, one would run BIGRED on each set, merge the true replicates within each set (discarding the erroneous samples), then combine the sets of merged samples before running BIGRED once more. By using a Poisson distribution to simulate AD data, we make the assumption that reads are uniformly distributed across the genome. While read data will often be more highly dispersed than these analyses, if at least *L* of the sites in those data have read depth of lambda or above, BIGRED will perform at least as well as in our analyses with these same parameters.

A motivation for BIGRED’s joint analysis framework is that pairwise-comparison methods might produce ambiguous results when more than two putative replicates exist, and we did, in fact, run into cases of this when applying the correlation method to real data. Of the cases where the method reported the presence of replicates when applying a replicate-call threshold of 0.85 and 0.80, 12.50 and 4.11% contained pairwise inconsistencies, respectively. By decreasing the call threshold, one lowers the number of ambiguous cases returned but doing so also increases the number of false positives returned. And although it may occur at low frequency, the possibility of pairwise inconsistencies exists and would be a problem for all methods that employ a pairwise-comparison approach.

## Conclusions

In this study, we introduced a statistical framework for detecting mislabeled and contaminated samples among putative replicates. Our method addresses key limitations of existing approaches and produced highly accurate results in simulation experiments even when applied to samples with low read depth. Our method is implemented as an R package called BIGRED, which is freely available for download: https://github.com/ac2278/BIGRED.

## Additional files

Additional file 1: A simplified representation of a VCF data file containing allele depth (AD) data for the *k* = 3 putative replicates of I011206. (PDF 55 kb)

Additional file 2: Names of the *k* putative replicates associated with each IITA genotype used in the study. (XLSX 37 kb)

Additional file 3: Names of the *k* putative replicates associated with each NaCRRI genotype used in the study. (XLSX 45 kb)

Additional file 4: Names of the *k* putative replicates associated with each NRCRI genotype used in the study. (XLSX 23 kb)

Additional file 5: Plot showing the number of source vectors associated with *k* for *k* ∈ {1, …, 8}. (PDF 95 kb)

Additional file 6: Samples removed from analysis in the comparison between BIGRED and hierarchical clustering due to low genome-wide mean read depth. (XLSX 36 kb)

Additional file 7: BIGRED’s accuracy as a function of the mean read depth of samples and the MAF of analyzed sites for *k* = 2 and *k* = 4. (PDF 80 kb)

Additional file 8: Additional plots for the simulation experiments outlined in “Simulation experiments to evaluate the impact of mean read depth and MAF on accuracy” for *S =* (1,2,1) and *S =* (1,2,3), showing the posterior probability assigned to all source vectors. (PDF 311 kb)

Additional file 9: Cases where the pairwise correlation method produced ambiguous results when applying a replicate-call threshold of 0.85. (PDF 38 kb)

Additional file 10: Cases where the pairwise correlation method produced ambiguous results when applying a replicate-call threshold of 0.80. (PDF 35 kb)

## Abbreviations

AD: Allelic depth
BIGRED: Bayes Inferred Genotype Replicate Error Detector
CPU: Central processing unit
GBS: Genotyping-by-sequencing
HTS: High-throughput sequencing
HWE: Hardy-Weinberg Equilibrium
IITA: International Institute of Tropical Agriculture
MAF: Minor allele frequency
NaCRRI: National Crops Resources Research Institute
NRCRI: National Root Crops Research Institute
PCA: Principal component analysis
REs: Restriction enzymes
VCF: Variant call format
WGS: Whole-genome sequence
