# Supplementary material for: A statistical framework for detecting mislabeled and contaminated samples using shallow-depth sequence data
Source: BMC Bioinformatics. 2018 Dec 12;19:478. doi: 10.1186/s12859-018-2512-8 (PMC6292093; doi:10.1186/s12859-018-2512-8)
Supplement: Supplementary file 1 — A simplified representation of a VCF data file containing allele depth (AD) data for the k = 3 putative replicates of I011206. (PDF 55 kb) [file 12859_2018_2512_MOESM1_ESM.pdf]

	$d = 1$	$d = 2$	$d = 3$
SNP 1	7,0	5,0	3,1
SNP 2	3,4	3,0	4,0
⋮	⋮	⋮	⋮
SNP M	4,0	5,1	7,0

A simplified representation of a VCF data file containing allele depth (AD) data for the k = 3 putative replicates of I011206.

In this example, we have shallow sequenced individual I011206 three different times using some HTS method. We obtained this output: a matrix whose rows represent polymorphic sites and whose columns represent the replicate sequence runs. The putative replicates are indexed with the variable d. Each element of the matrix consists of two (comma-separated) integers, representing the observed counts for allele A and B. We wish to determine whether the DNA samples from these three sequence runs originate from one individual.
