# Supplementary material for: A statistical framework for detecting mislabeled and contaminated samples using shallow-depth sequence data
Source: BMC Bioinformatics. 2018 Dec 12;19:478. doi: 10.1186/s12859-018-2512-8 (PMC6292093; doi:10.1186/s12859-018-2512-8)

The plot below shows the number of source vectors associated with k for $k \in \{1, \ldots, 8\}$. The number of possible source vectors increases exponentially as k increases. For this reason, we do not recommend using BIGRED on cases where k>7 but do not anticipate many scenarios where a researcher would have sequenced a given individual more than seven times. If this scenario does occur, we discuss possible solutions in the Discussion of the main text.

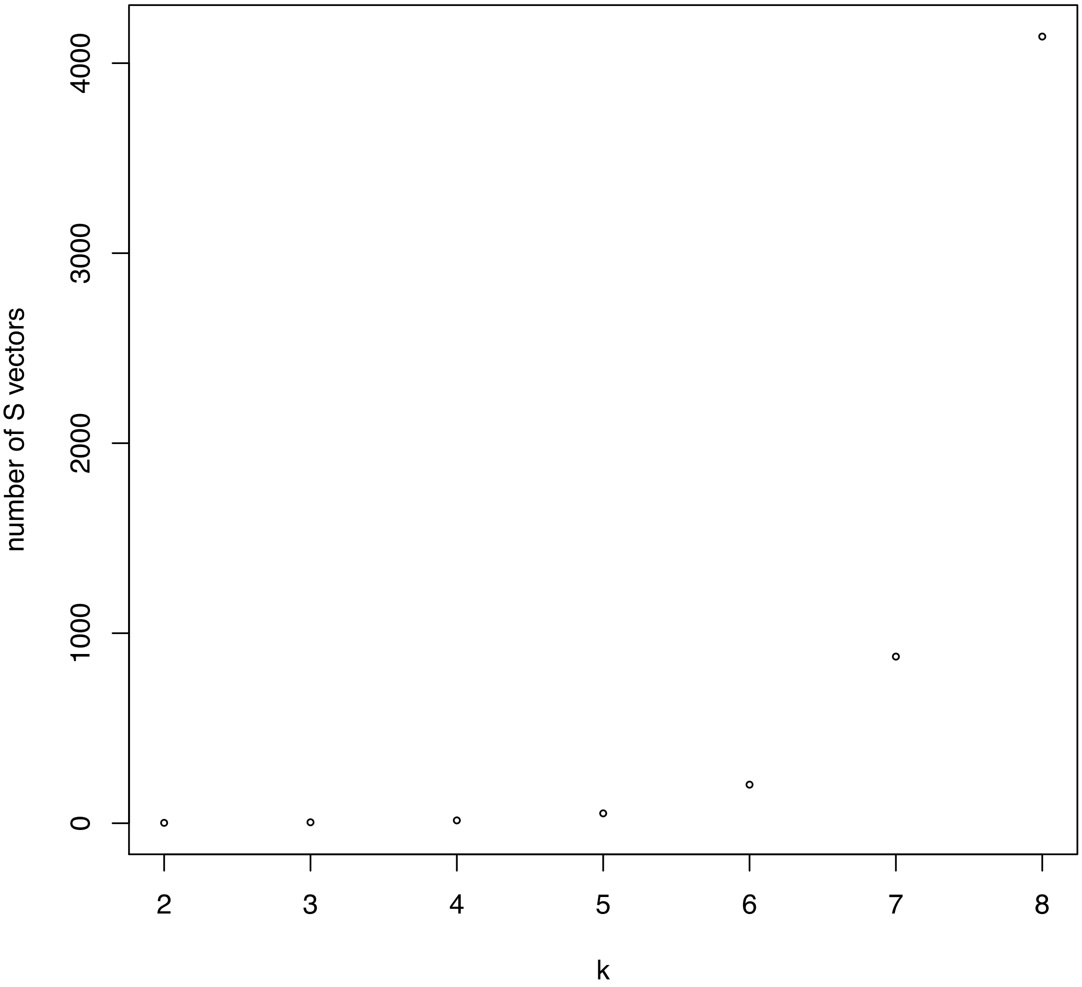

Supplement: Supplementary file 5 — Plot showing the number of source vectors associated with k for k ∈ {1, …, 8}. (PDF 95 kb) [file 12859_2018_2512_MOESM5_ESM.pdf]
