# Supplementary material for: A statistical framework for detecting mislabeled and contaminated samples using shallow-depth sequence data
Source: BMC Bioinformatics. 2018 Dec 12;19:478. doi: 10.1186/s12859-018-2512-8 (PMC6292093; doi:10.1186/s12859-018-2512-8)
Supplement: Supplementary file 7 — BIGRED’s accuracy as a function of the mean read depth of samples and the MAF of analyzed sites for k = 2 and k = 4. (PDF 80 kb) [file 12859_2018_2512_MOESM7_ESM.pdf]

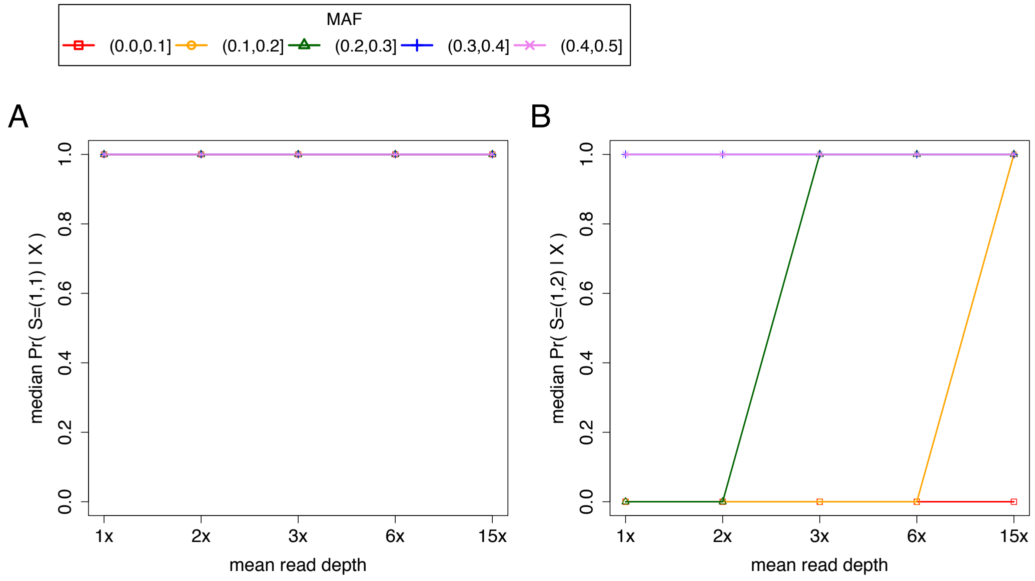

Algorithm's accuracy as a function of the mean read depth of samples and the MAF of analyzed sites for *k* = 2.

(A and B) Each plot shows estimates of the median posterior probability of the true source vector (*y*-axis) as a function of mean read depth of samples (*x*-axis) and MAF of sites (legend). Each data point presents the median posterior probability of S = (1,1) and S = (1,2) across 15 and 100 runs of the algorithm, respectively.

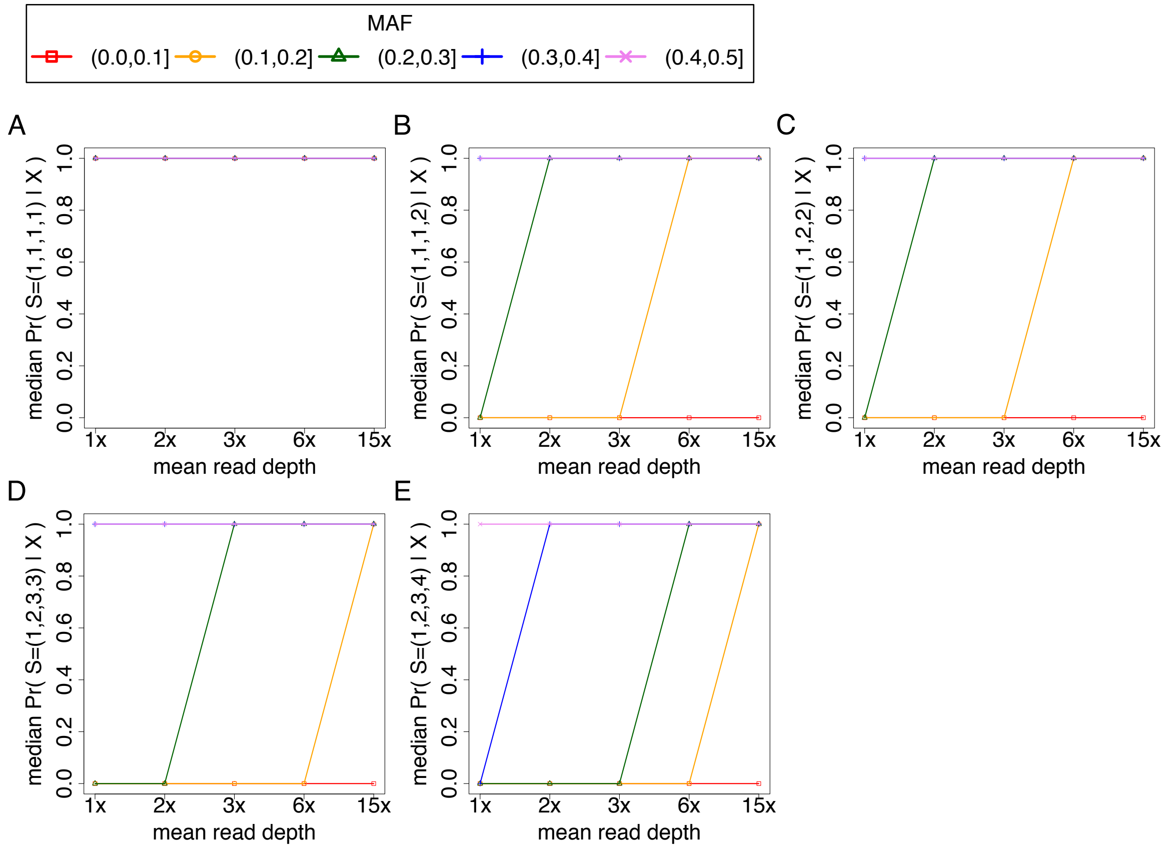

Algorithm's accuracy as a function of the mean read depth of samples and the MAF of analyzed sites for *k* = 4.

(A, B, C, D, and E) Each plot shows estimates of the median posterior probability of the true source vector (*y*-axis) as a function of mean read depth of samples (*x*-axis) and MAF of sites (legend). Each data point presents the median posterior probability of S = (1,1,1,1), S = (1,1,1,2), S = (1,1,2,2), S = (1,2,3,3), and S = (1,2,3,4) across 15, 100, 100, 100, and 100 runs of the algorithm, respectively.
