# Supplementary material for: A statistical framework for detecting mislabeled and contaminated samples using shallow-depth sequence data
Source: BMC Bioinformatics. 2018 Dec 12;19:478. doi: 10.1186/s12859-018-2512-8 (PMC6292093; doi:10.1186/s12859-018-2512-8)
Supplement: Supplementary file 8 — Additional plots for the simulation experiments outlined in “Simulation experiments to evaluate the impact of mean read depth and MAF on accuracy” for S = (1,2,1) and S = (1,2,3), showing the posterior probability assigned to all source vectors. (PDF 311 kb) [file 12859_2018_2512_MOESM8_ESM.pdf]

Additional plots for the simulation experiments outlined in "Simulation experiments to evaluate the impact of mean read depth and MAF on accuracy" for *S* = (1,2,1) and *S* = (1,2,3), showing the posterior probability assigned to all source vectors.

We present a series of four plots for the experiments where we simulated *S* = (1,2,1) and another four plots for the experiments where we simulated *S* = (1,2,3). Each plot consists of five subplots (one subplot for each of the tested mean read depths or lambdas). The title of each subplot shows the true (simulated) source vector for that experiment, the mean depth of putative replicates, and the MAF of sampled sites. Each subplot consists of five boxplots (one boxplot for each of the five possible source vectors). Each boxplot consists of 100 data points. We excluded plots for the (0.0,0.1] MAF interval since non zero probabilities were assigned only to *S*=(1,1,1) at every lambda. These plots reiterate the behavior observed in Figure 6 (main text) but do so at a higher resolution. For a given MAF interval, with the exception of (0.0,0.1], BIGRED shifts the probability away from *S*=(1,1,1) towards the true (simulated) source vector as the mean read depth of samples increases.

Plots for *S* = (1,2,1):

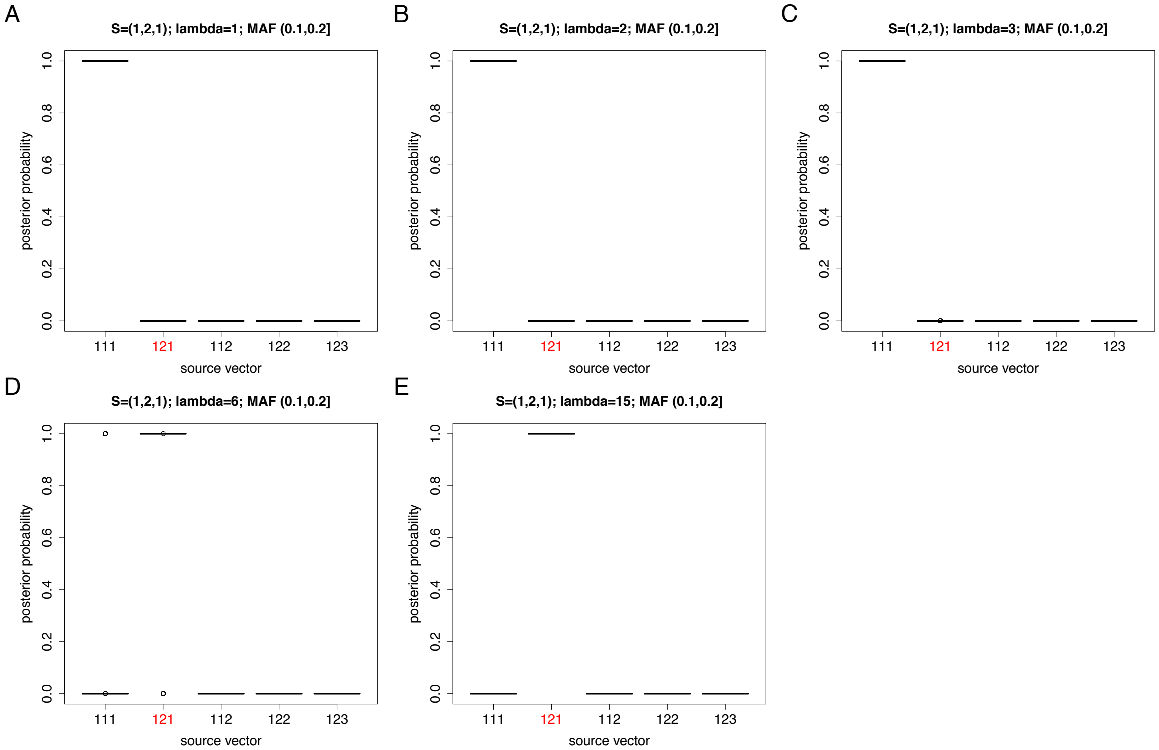

Plots where *S* = (1,2,1) was the true (simulated) source vector and sites were sampled from the (0.1,0.2] MAF interval.

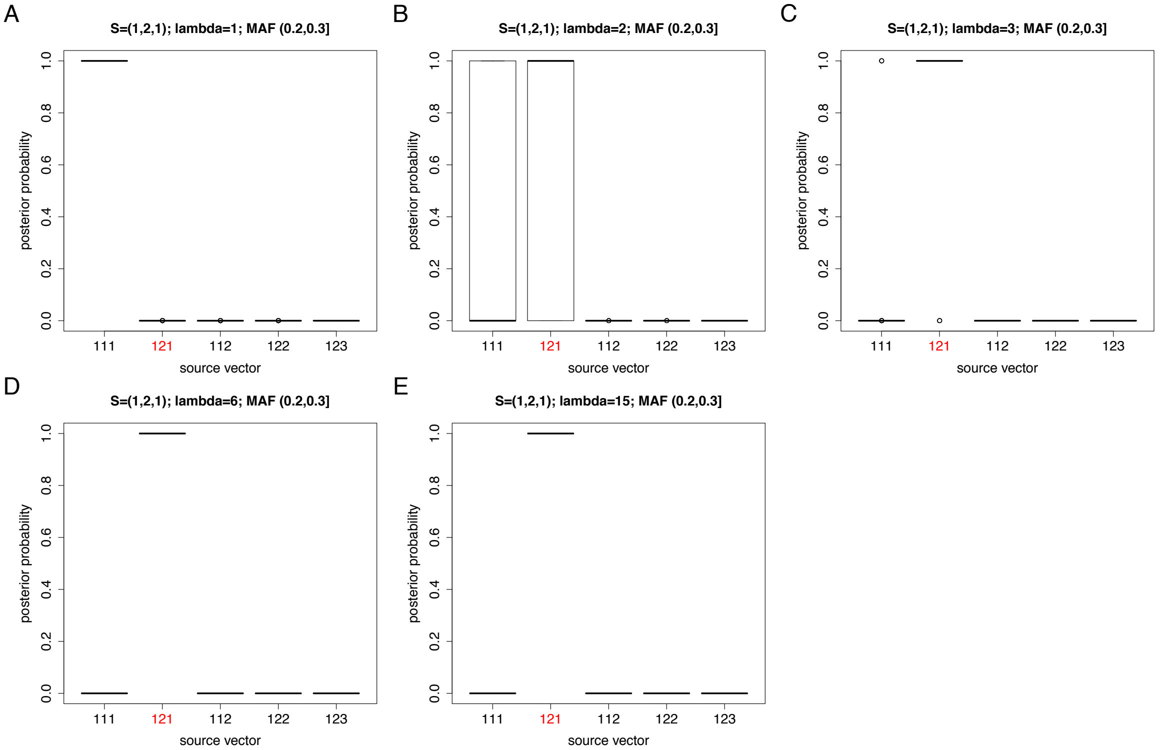

Plots where S = (1,2,1) was the true (simulated) source vector and sites were sampled from the (0.2,0.3] MAF interval.

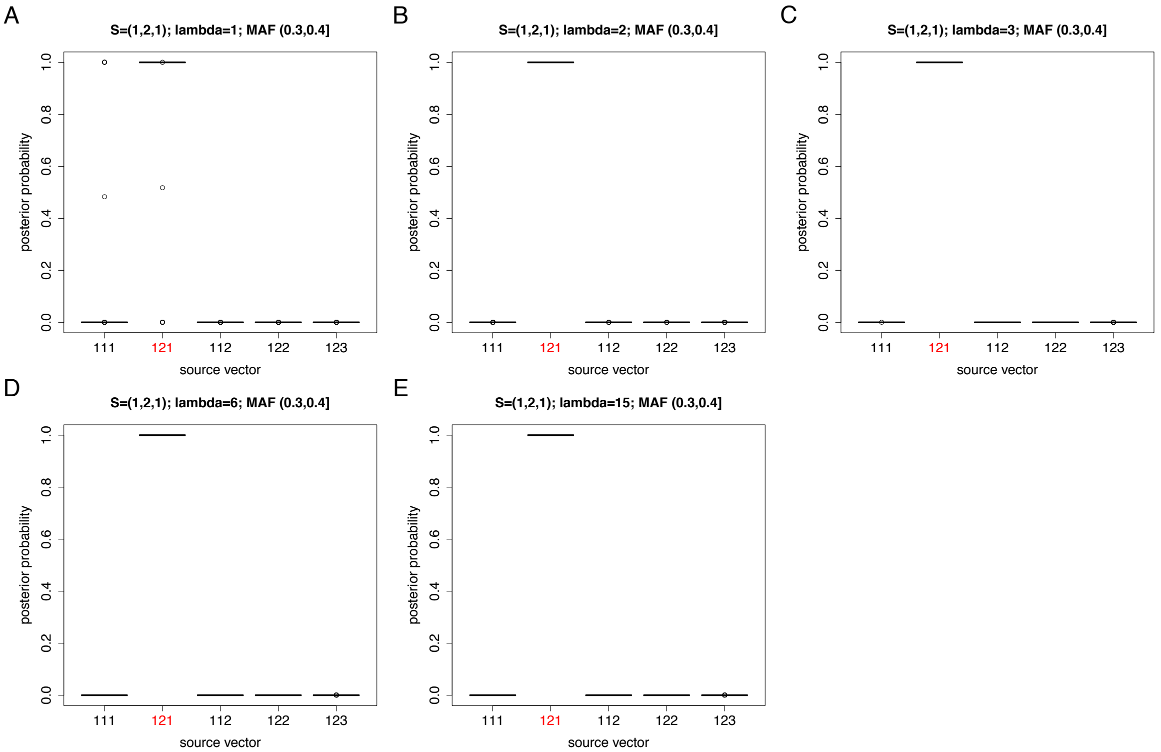

Plots where S = (1,2,1) was the true (simulated) source vector and sites were sampled from the (0.3,0.4] MAF interval.

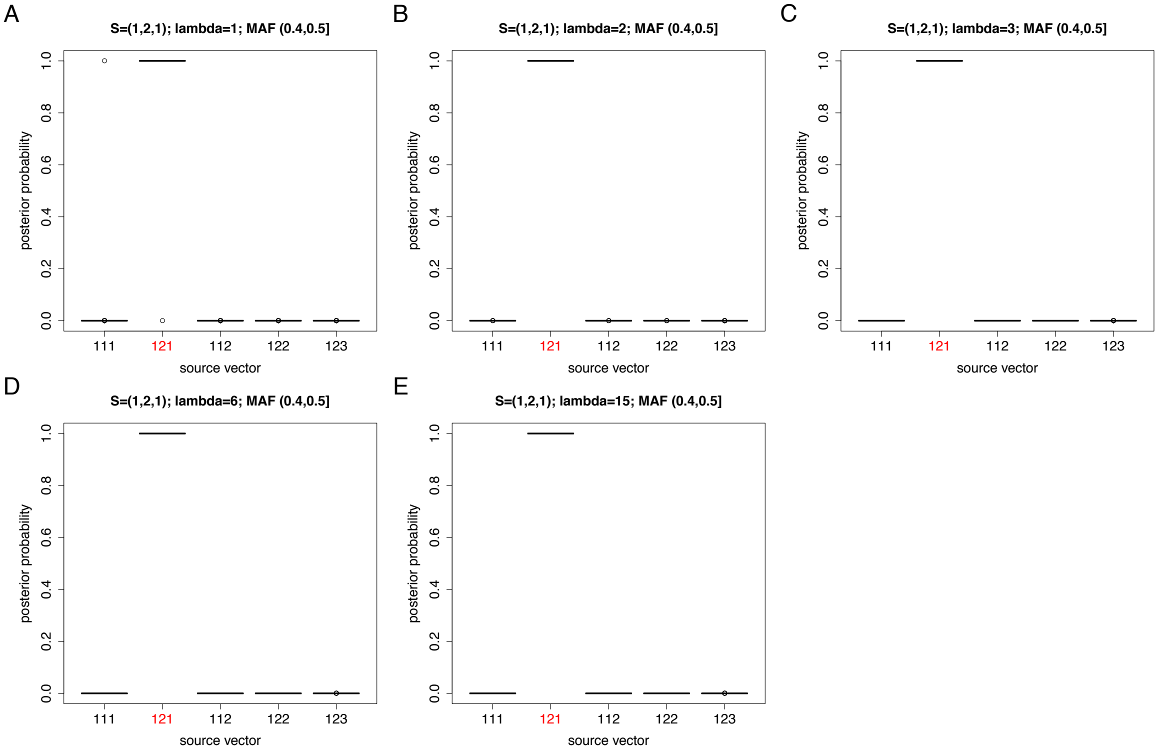

Plots where *S* = (1,2,1) was the true (simulated) source vector and sites were sampled from the (0.4,0.5] MAF interval.

Plots for S = (1,2,3):

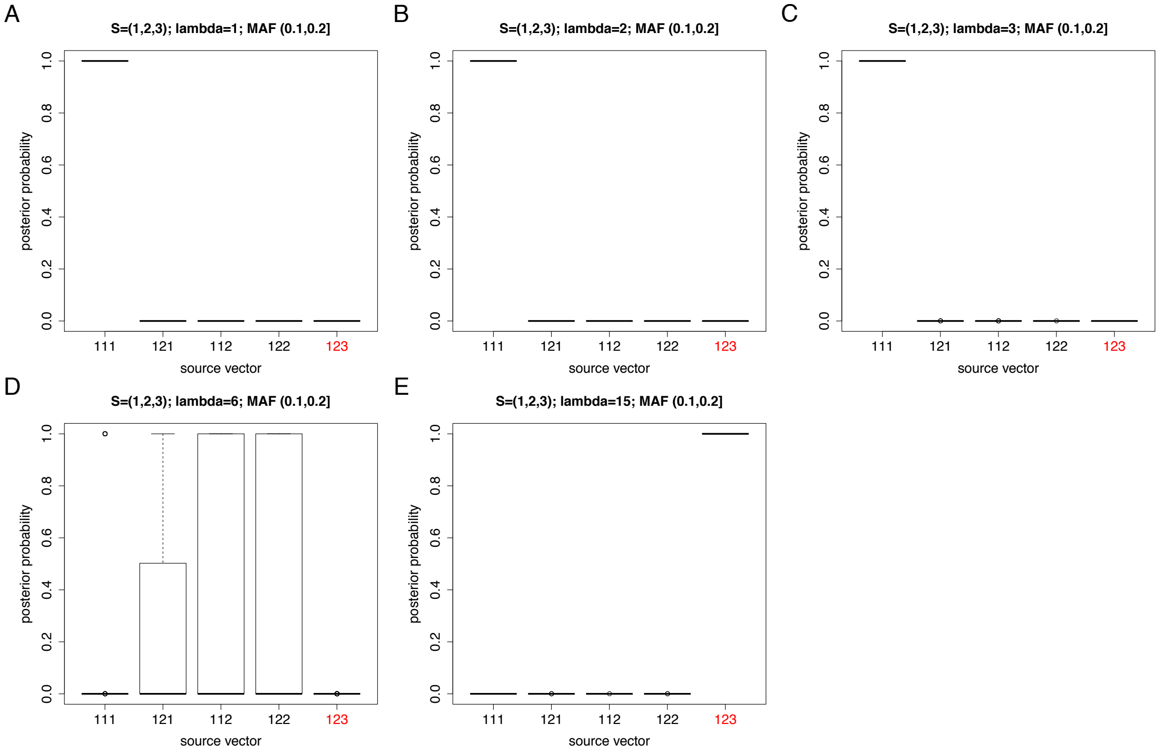

Plots where S = (1,2,3) was the true (simulated) source vector and sites were sampled from the (0.1,0.2] MAF interval.

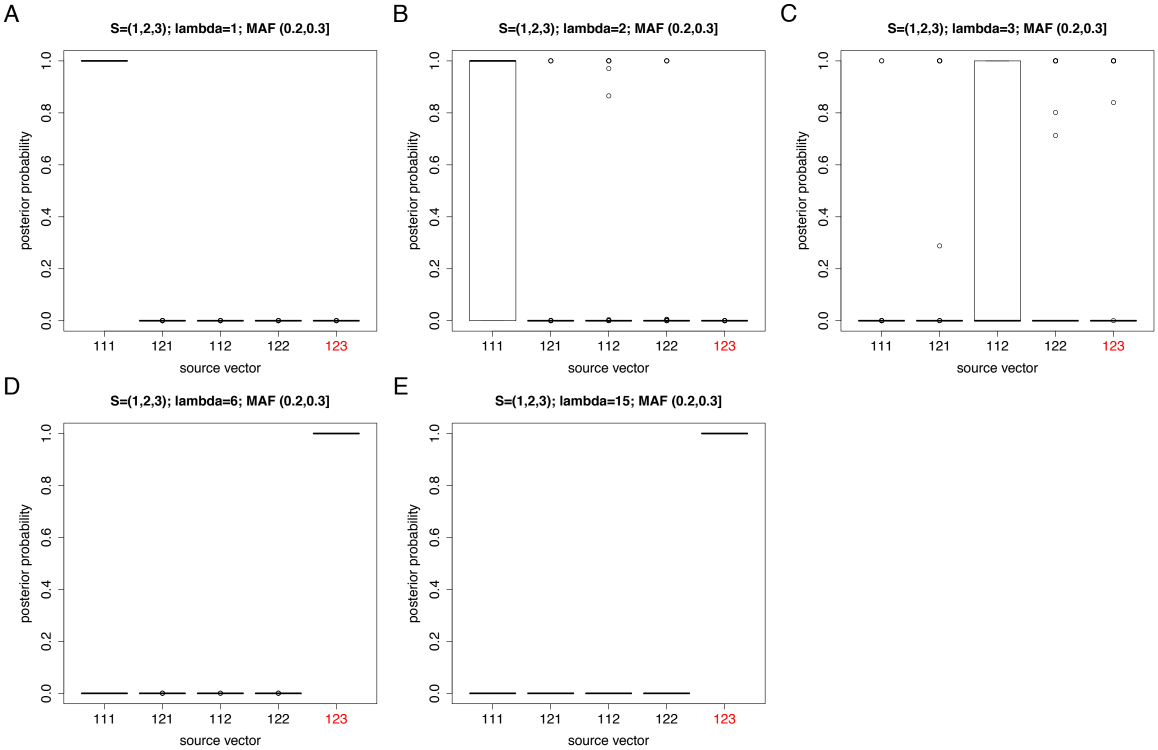

Plots where S = (1,2,3) was the true (simulated) source vector and sites were sampled from the (0.2,0.3] MAF interval.

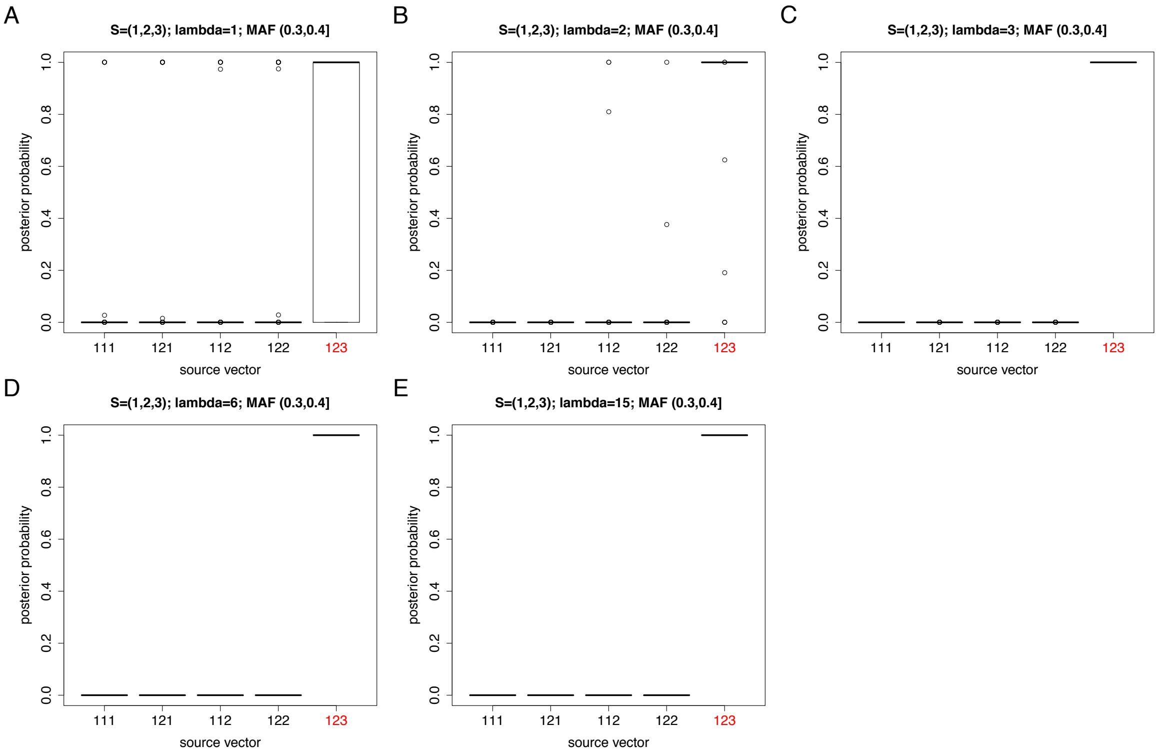

Plots where S = (1,2,3) was the true (simulated) source vector and sites were sampled from the (0.3,0.4] MAF interval.

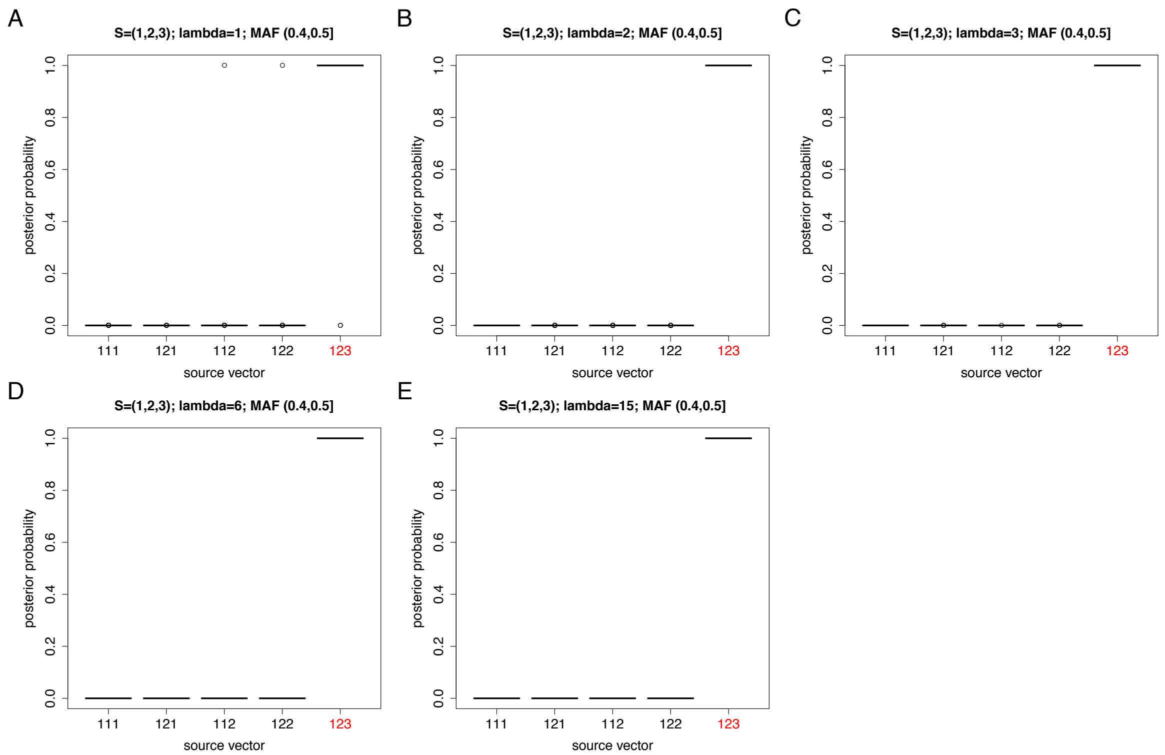

Plots where *S* = (1,2,3) was the true (simulated) source vector and sites were sampled from the (0.4,0.5] MAF interval.
