# Supplementary material for: A statistical framework for detecting mislabeled and contaminated samples using shallow-depth sequence data
Source: BMC Bioinformatics. 2018 Dec 12;19:478. doi: 10.1186/s12859-018-2512-8 (PMC6292093; doi:10.1186/s12859-018-2512-8)
Supplement: Supplementary file 9 — Cases where the pairwise correlation method produced ambiguous results when applying a replicate-call threshold of 0.85. (PDF 38 kb) [file 12859_2018_2512_MOESM9_ESM.pdf]

When examining cases of *k*=3 and using a replicate-call threshold of 0.85, we found 80 cases (out of 154) where the pairwise correlation method awarded any pair of samples (of an individual) replicate status. Of these 80 cases, we found 10 cases where the method produced ambiguous results. We list the results of these 10 cases below.

M980004

	M980004:250300454	M980004:250249819	M980004:250164026
M980004:250300454	1.0000000	0.8532322	0.8324890
M980004:250249819	0.8532322	1.0000000	0.8565424
M980004:250164026	0.8324890	0.8565424	1.0000000

MM970016

	MM970016:250300474	MM970016:250249824	MM970016:250164029
MM970016:250300474	1.0000000	0.8558827	0.8395040
MM970016:250249824	0.8558827	1.0000000	0.8633336
MM970016:250164029	0.8395040	0.8633336	1.0000000

I050128

	I050128:250300204	TMS050128:250399901	TMS050128:250107908
I050128:250300204	1.0000000	0.8249625	0.8539455
TMS050128:250399901	0.8249625	1.0000000	0.8613728
TMS050128:250107908	0.8539455	0.8613728	1.0000000

MM970646

	MM970646:250300486	MM970646:250302430	MM970646:250249827
MM970646:250300486	1.0000000	0.8542486	0.8733996
MM970646:250302430	0.8542486	1.0000000	0.8489665
MM970646:250249827	0.8733996	0.8489665	1.0000000

I9102324

	I9102324:250399839	I9102324:250090766	TMS9102324:250134500
I9102324:250399839	1.0000000	0.8725842	0.8196078
I9102324:250090766	0.8725842	1.0000000	0.8591781
TMS9102324:250134500	0.8196078	0.8591781	1.0000000

TMS13F1332P0045

	TMS13F1332P0045:250301854	2013_10332_45:250251328	2013_0332_45:250161118
TMS13F1332P0045:250301854	1.0000000	0.8554351	0.8680323
2013_10332_45:250251328	0.8554351	1.0000000	0.8443854
2013_0332_45:250161118	0.8680323	0.8443854	1.0000000

TMS13F1034P0002

	TMS13F1034P0002:250302115	2013_10034_2:250251352	2013_10034_2:250164201
TMS13F1034P0002:250302115	1.0000000	0.8454982	0.8571305
2013_10034_2:250251352	0.8454982	1.0000000	0.8585334
2013_10034_2:250164201	0.8571305	0.8585334	1.0000000

TMS13F1024P0002

	TMS13F1024P0002:250303528	2013_10024_2:250251351
TMS13F1024P0002:250303528	1.0000000	0.8601510
2013_10024_2:250251351	0.8601510	1.0000000
2013_0024_2:250162894	0.8659588	0.8344528

	2013_0024_2:250162894
TMS13F1024P0002:250303528	0.8659588
2013_10024_2:250251351	0.8344528
2013_0024_2:250162894	1.0000000

TMS13F1160P0003

	TMS13F1160P0003:250303661	2013_0160_3:250160398
TMS13F1160P0003:250303661	1.0000000	0.8660223
2013_0160_3:250160398	0.8660223	1.0000000
2013_10160_3:250251245	0.8537107	0.8307236

	2013_10160_3:250251245
TMS13F1160P0003:250303661	0.8537107
2013_0160_3:250160398	0.8307236
2013_10160_3:250251245	1.0000000

TMS13F1018P0005

	TMS13F1018P0005:250301197	2013_0018_5:250162314
TMS13F1018P0005:250301197	1.0000000	0.8613214
2013_0018_5:250162314	0.8613214	1.0000000
2013_10018_5:250251187	0.8565490	0.8483976

	2013_10018_5:250251187
TMS13F1018P0005:250301197	0.8565490
2013_0018_5:250162314	0.8483976
2013_10018_5:250251187	1.0000000
