# Supplementary material for: A statistical framework for detecting mislabeled and contaminated samples using shallow-depth sequence data
Source: BMC Bioinformatics. 2018 Dec 12;19:478. doi: 10.1186/s12859-018-2512-8 (PMC6292093; doi:10.1186/s12859-018-2512-8)
Supplement: Supplementary file 10 — Cases where the pairwise correlation method produced ambiguous results when applying a replicate-call threshold of 0.80. (PDF 35 kb) [file 12859_2018_2512_MOESM10_ESM.pdf]

When examining cases of k=3 and using a replicate-call threshold of 0.80, we found 146 cases (out of 154) where the pairwise correlation method awarded any pair of samples (of an individual) replicate status. Of these 146 cases, we found six cases where the method produced ambiguous results. We list the results of these six cases below.

I082425

	I082425:250399888	I082425:250099292	I082425:250304667
I082425:250399888	1.0000000	0.8133687	0.8530645
I082425:250099292	0.8133687	1.0000000	0.7951555
I082425:250304667	0.8530645	0.7951555	1.0000000

I011086

	I011086:250300303	I011086:250099231	TMS011086:250107973
I011086:250300303	1.0000000	0.8077456	0.8271401
I011086:250099231	0.8077456	1.0000000	0.7614757
TMS011086:250107973	0.8271401	0.7614757	1.0000000

TMS13F1111P0012

	TMS13F1111P0012:250301853	2013_0111_12:250160049
TMS13F1111P0012:250301853	1.0000000	0.8475052
2013_0111_12:250160049	0.8475052	1.0000000
2013_10111_12:250251234	0.7933040	0.8046933

	2013_10111_12:250251234
TMS13F1111P0012:250301853	0.7933040
2013_0111_12:250160049	0.8046933
2013_10111_12:250251234	1.0000000

TMS13F1109P0007

	TMS13F1109P0007:250301722	2013_0109_7:250160028	2013_10109_7:250251231
TMS13F1109P0007:250301722	1.0000000	0.8472932	0.7968483
2013_0109_7:250160028	0.8472932	1.0000000	0.8109981
2013_10109_7:250251231	0.7968483	0.8109981	1.0000000

TMS13F1109P0009

	TMS13F1109P0009:250465368	TMS13F1109P0009:250302119	2013_0109_9:250160030
TMS13F1109P0009:250465368	1.0000000	0.7981975	0.8351301
TMS13F1109P0009:250302119	0.7981975	1.0000000	0.8229579
2013_0109_9:250160030	0.8351301	0.8229579	1.0000000

TMS13F1063P0007

	TMS13F1063P0007:250302247	2013_10063_7:250251355	2013_10063_7:250164227
TMS13F1063P0007:250302247	1.0000000	0.7924421	0.8400863
2013_10063_7:250251355	0.7924421	1.0000000	0.8103049
2013_10063_7:250164227	0.8400863	0.8103049	1.0000000
